# Does Pre-Procedural Anxiety Affect the Consumption of Sedatives During Colonoscopy?

**DOI:** 10.5152/TJAR.2022.22130

**Published:** 2023-02-01

**Authors:** Başak Ceyda Meço, Cihangir Akyol, Ali Abbas Yılmaz, Helin Şahintürk, Mehmet Ayhan Kuzu

**Affiliations:** 1 Department Anaesthesiology and Intensive Care Medicine, Ankara University Faculty of Medicine, Ankara, Turkey; 2 Department of General Surgery, Ankara University Faculty of Medicine, Ankara, Turkey

**Keywords:** Anaesthesia, anxiety, colonoscopy, outpatients

## Abstract

**Objective:**

Anxiety is an unpleasant emotional state with systemic effects. The anxiety level of the patients may increase the requirements for sedation during colonoscopy. The aim of the study was to evaluate the effect of pre-procedural anxiety on the dose of propofol.

**Methods:**

A total of 75 patients undergoing colonoscopy were enrolled in the study. Patients were informed about the procedure and their anxiety levels were assessed. The level of sedation was defined as a bispectral index of 60 and was achieved by target-controlled infusion of propofol. Patients’ characteristics, haemodynamic profiles, anxiety levels, propofol dosage, and complications were recorded. The procedure duration, difficulty score for colonoscopy assessed by the surgeon, and the patient’s and surgeon’s satisfaction with sedation instrument scores were recorded.

**Results:**

A total of 66 patients were studied. The anxiety scores were not correlated with the total propofol dosage, haemodynamic parameters, the time needed to reach a bispectral index value of 60, surgeon and patient satisfaction, and the time needed to regain consciousness. No complications were observed.

**Conclusions:**

In patients receiving deep sedation for elective colonoscopies, the pre-procedural anxiety level is not related to sedative requirement, post-procedural recovery, or surgeon and patient satisfaction.

**Main Points**

- The anxiety scores were not correlated with the total propofol dosage, haemodynamic parameters, the time needed to reach a bispectral index value of 60, surgeon and patient satisfaction, and the time needed to regain consciousness.
- There were no correlations between the total propofol dosage (adjusted for time and weight) and Spielberger State Anxiety Inventory and Spielberger Trait Anxiety Inventory levels. Our patient population was informed by a well-experienced nurse who was trained in colonoscopy. This might have decreased the pre-procedural anxiety levels in our patients, which might in turn have influenced the correlation between anxiety and sedation.
- We evaluated a cohort with relatively low levels of anxiety, possibly due to good pre-procedural information. This anxiety level might have decreased the need for sedation, which might have influenced the correlations between anxiety and sedation-related parameters.

## Introduction

Preoperative anxiety is an unpleasant emotional state of discomfort and is characterised by subjective feelings of nervousness, tension, and worry. Anxiety can also be described according to individual differences as proneness as a personality trait (trait anxiety). On the other hand, state anxiety can be defined as the subjective feelings of nervousness and tension when one is subjected to an anxiety-provoking stimulus (state anxiety).^1^ Consequently, the level of trait anxiety of an individual results in differences in state anxiety between people when exposed to stressful situations.

It is well known that the preoperative period for any procedure is a high-level stressful situation.^2^ Subsequently, the anxiety level of patients during the preoperative period may be high and may lead to increased levels of catecholamines, resulting in unwanted haemodynamic and metabolic responses. This anxiety can also worsen patients’ perception of discomfort, pain, and worry.^3^

Colonoscopy is an outpatient procedure not only performed as a screening test but also for diagnosis and treatment of a wide range of gastrointestinal problems. It is often viewed as an invasive procedure with the potential for embarrassment, discomfort, and worry.^4^ Such fears can result in a high level of state anxiety that may decrease the tolerance and cooperation of the patient, limit the success of the procedure, increase the likelihood of complications, and increase the requirements for sedation and analgesia.^3^ Therefore, colonoscopy is frequently performed under deep sedation to prevent patient discomfort, enhance tolerance, decrease the procedure duration, and increase the success rate. The level of sedation is critical for patient comfort and the success of the procedure. Also, the duration of the procedure is becoming a more and more important parameter these days. For such discomforting procedures, patients often describe ideal sedation as deep amnesia during the procedure. On the other hand, anaesthesiologists highlight the importance of safety and complete cognitive recovery at the end of the procedure, while the physicians who performed colonoscopy prioritise the ease, rapidity, and effectiveness of the procedure.^5^ Also, as stated previously, the trait anxiety level of the patient before the procedure may increase the requirement for sedation. It is important for the anaesthesiologist to predict the effective dosages of sedation before the procedure to ensure rapid and comfortable sedation as well as prompt recovery and discharge of the patient. Therefore, the trait and state anxiety levels of patients should be considered as one of the most important parameters to assess and take into consideration during the preparation of such discomforting and sometimes painful procedure.

The aim of this prospective cohort study was to evaluate the pre-procedural anxiety levels of patients and to evaluate their effect on the dose of propofol needed to achieve the required sedation levels.

## Methods

### Inclusion Criteria

After obtaining Institutional Review Board approval from the Ankara University Faculty of Medicine with the approval number 10-306-12 and written informed consent that patients were included in an observational study, 75 American Society of Anesthesiologists (ASA) I-II patients 18-65 years old undergoing elective colonoscopy for screening (patients over the age of 50 and patients with a family history of colorectal cancer or colorectal adenoma) were enrolled in the study. This study was registered to clinicaltrial.gov with the registration number NCT 01958151.

### Exclusion Criteria

Patients with a history of colonic resection or any intraabdominal surgery, a prescription of anxiolytic medications, and a predicted allergy to propofol were not studied. Also, patients refusing sedation were not included in the study.

### Measurements

All patients were instructed to take a standard colon preparation regimen before the procedure. On the day of the first visit to the surgeon, after the decision of colonoscopy, all patients were informed by the surgeon, related to the planned procedure. Also, patients were sent home with a brochure explaining the basic procedures.

On the day of the procedure, demographic data were collected and all patients were informed about the procedure, one more time, by a well-experienced nurse who was trained in colonoscopy. Patients’ questions related to the procedure or anaesthesia were answered and afterwards, they were asked to complete the Spielberger State-Trait Anxiety Inventory (STAI) in an isolated, calm room before being transferred to the procedure room.^6^

The STAI was developed by Spielberger et al^7^ in 1970 and is designed to measure temporary and situational anxiety as well as the tendency to show situational anxiety under stress.^5-8^ It is a reliable and sensitive measure of anxiety in adults. The STAI consists of 40 self-reported items that measure state and trait anxiety scores ranging from 20 to 80, with higher scores indicating higher levels of anxiety.^6-10^ The Trait Portion (20 items) measures a person’s general disposition and proneness to anxiety, including general states of calmness and security (STAI-T), and the State Portion (20 items) measures how a person feels at the time of the operation using subjective feelings like nervousness, worry, and arousal (STAI-S).^6^ The instrument is rated on a 4-point scale, with scores summed to obtain the overall score.

It is simple to use, generally taking <5 minutes to complete, and is easy to score.^6,9^ The range of scores for each subtest is 20-80 with the higher score indicating greater anxiety. A cut point of 39-40 is defined as a clinically significant STAI-S score. However, other studies have suggested a higher cut-off of 54-55 for older adults.^6^

Upon the arrival of the patient in the procedure room, the patient was one more time informed related to the procedure by the surgeon who will perform it. Also, the anaesthesiologist has given information related to the monitorised anaesthesia care and sedation to the patient. Then, all patients were monitored with routine monitoring, and the bispectral index (BIS: BIS A-1050 Monitor; Aspect Medical Systems, Newton, Mass, USA) was used to evaluate the depth of sedation. A BIS sensor was placed on the forehead of the patient and connected to the BIS monitor. No premedication was employed, and all data were collected by an anaesthetist (H.S.) blinded to the STAI scores and sedation procedures.

Oxygen was administered at 4 L min^−1^ via a facemask to maintain peripheral oxygen saturation (SpO_2_) above 95% and the patient was placed in the lateral decubitus position. The baseline heart rate, mean arterial pressure (MAP), SpO_2_, and BIS values were recorded, and 2% propofol infusion was started via an effect-site target-controlled infusion system (Marsh model) to a preset target concentration of 3.5 μg mL^−1^ until the patient reached the desired level of sedation as determined by BIS value of 60.^11,12^ If the BIS index did not reach the defined level within 3 minutes, the target concentration was increased in increments of 0.2 μg mL^−1^. The procedure commenced upon the achievement of a BIS value of 60. Thereafter, the sedation level was guided with BIS monitoring, aiming toward a BIS value of 60-70. The time to reach the required BIS value was recorded. If cardiopulmonary depression was observed, the propofol dose was decreased by 0.2 μg mL^−1^. The jaw thrust manoeuvre was performed if there were any signs of airway obstruction or respiratory depression. In the event of hypoxemia (SpO_2_ <90%), positive pressure ventilation was performed and ephedrine was administered if MAP decreased by more than 20% of the baseline value.

All colonoscopies were performed by 1 of 2 surgeons who were also blinded to STAI scores (C.A., M.A.K.). A video-endoscopy system (Fujinon 490ZW5; Fujinon Corp., Saitama, Japan) was used. In this study, caecal intubation and extubation were considered as successful colonoscopy.

During the procedure, haemodynamic parameters, the total propofol dosage required to reach the appropriate BIS values, and complications (respiratory depression, need for ventilation, nausea, vomiting, laryngospasm, and agitation) were recorded. The procedure duration, difficulty score for colonoscopy (0 = easy; 10 = very difficult) assessed by the surgeon after the procedure, and surgeon satisfaction with sedation instrument scores were recorded.^13^

After the patient had recovered fully, the time needed to regain consciousness assessed by a Modified Aldrete Score ≥9 after discontinuing the infusion pump was recorded and the patient satisfaction with sedation instrument scores was evaluated.^13,14^

The main association that we examined was between the pre-procedure anxiety as measured by using the STAI scores and total propofol dosage used to reach a BIS value of 60. Based on our preliminary data, we presumed a correlation coefficient of 0.25. We needed at least 60 patients to set a significance level of .05 (2-sided) and achieve a power of 0.80. To compensate for possible dropouts, we enrolled 75 patients.

### Statistical Analysis

Data are expressed as means ± SD and numbers of patients. Anxiety scores and drug consumption were assessed by univariate variance analysis. The chi-square test was used for categorical data. The association between pre-procedure anxiety and the total propofol dosage, haemodynamic parameters, the time needed to reach a BIS value of 60, the number of target controlled infusion (TCI) interventions, surgeon satisfaction with sedation instrument scores, patient satisfaction with sedation instrument scores, and the time needed to regain consciousness assessed by a Modified Aldrete Score ≥9 after discontinuing the infusion pump were assessed by Spearman correlation coefficient. All statistical analyses were performed using Statistical Package for the Social Sciences version 15.0 (SPSS Inc., Chicago, IL, USA). In all analyses, *P* < .05 was taken to indicate statistical significance.

## Results

**Figure 1.**
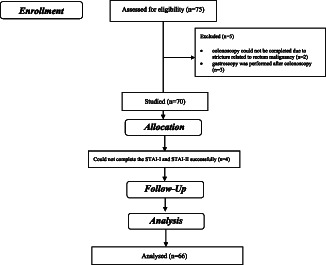
Overview of the cohort studied.

A total of 75 patients were enrolled in the study. In 2 patients, colonoscopy could not be completed due to stricture related to rectum malignancy. Four patients could not complete the STAI-S and STAI-T successfully, and gastroscopy was performed after colonoscopy in 3 patients. Therefore, a total of 9 patients were excluded from the study. Finally, the data for 34 females and 32 males were included in the statistical analysis (Figure 1). The patients’ characteristics and procedure-related parameters are shown in Table 1. Anaesthesia-related parameters are presented in Table 2.

There were no correlations between the total propofol dosage (adjusted for time and weight) and STAI-S and STAI-T levels. There were no differences in TCI intervention and propofol doses. Propofol doses and TCI intervention numbers according to STAI-S levels are given in Table 3. Propofol doses were similar according to STAI-T (*P* = .2)

Additional analyses were performed to identify correlations between STAI scores and baseline haemodynamic parameters, the time needed to reach a BIS value of 60, surgeon satisfaction with sedation instrument scores, patient satisfaction with sedation instrument scores, and the time needed to regain consciousness assessed by a Modified Aldrete Score ≥9 after discontinuing the infusion pump. However, no correlations were found in these comparisons.

All procedures were completed without any procedure-related complications. The caecal intubation rate was 86.3%. None of the patients required assisted ventilation. The procedure duration for colonoscopy and the anaesthesia time were 16.5 ± 6.7 minutes and 21.8 ± 81 minutes, respectively.

## Discussion

The results of this study indicated no association between pre-procedural anxiety level assessed before the procedure with STAI scores and sedative requirements for deep sedation during colonoscopy procedures. In addition, haemodynamic parameters, the time needed to reach a BIS value of 60, surgeon and patient satisfaction, and the time needed to regain consciousness were not correlated with pre-procedural STAI scores.

Several studies have investigated the effects of pre-procedural anxiety on anaesthetic requirements during sedation and general anaesthesia.^15-17^ Hong et al^17^ evaluated pre-procedural anxiety with Visual Analogue Scale (VAS) score and titrated the propofol dosage with sedation scale and clinical indicators. They demonstrated that higher anxiety levels were associated with higher total propofol dosage for conscious sedation. In another study, the dosage of propofol was correlated with STAI score during light to moderate levels of sedation (BIS: 75-85), but it was correlated with trait anxiety only during deeper sedation (BIS: 65).^18^ However, Maranets and Kain^16^ reported that during general anaesthesia practice, STAI-S levels before surgery were not correlated with intraoperative anaesthetic requirements. Also, in another study, the pre-procedural anxiety was assessed using the Beck score and no correlation was found between pre-procedural anxiety and sedative requirements for deep sedation in patients undergoing colonoscopy.^19^ These results anticipated that the effects of anxiety on propofol dosage might be lower when the level of sedation is deepened.

The results of the present study are in agreement with previous reports of the association of pre-procedural anxiety with deep sedation or general anaesthesia. This may be explained by the level of sedation suppressing the effects of anxiety.

The level of sedation is frequently measured based on subjective observations and clinical scoring methods, such as the observer’s assessment of alertness/sedation. It can be difficult to maintain a constant level of sedation using these subjective methods. On the other hand, BIS analysis has been shown to be effective in assessing the level of responsiveness of patients and to be sensitive for predicting the loss of consciousness. In several studies, BIS analysis showed a good correlation with the clinically observed sedation level.^19^ Bispectral index monitoring seems to be a more objective and accurate means of monitoring sedation, which allows comparison of the drug dosages necessary to achieve the same level of sedation.

In this study, the sedation level was followed up with BIS, and the target BIS level was 60-70. Therefore, all patients were maintained at a similar level of sedation (deep sedation); this might have suppressed the effects of anxiety in all patients in an identical manner.

In a cohort of 98 patients, Ersöz et al^9^ defined a pre-procedural STAI-S score of 45.7 ± 10.2 for gastrointestinal endoscopy and an STAI-S score of 44.8 ± 10.1 for colonoscopy and concluded that these diagnostic procedures were associated with remarkable anxiety in patients. In another study, Arabul et al^20^ examined the effects of an information video on the anxiety level in 227 patients undergoing colonoscopy and reported STAI-S levels of 40.5 ± 50.4 and 45 ± 5.9 in the group who watched the information video and in the group who were informed verbally, respectively. The mean STAI-S in our cohort was 33.8 ± 8.7, which was lower than in these other studies performed in similar patient populations. Parker et al^21^ showed in their study that patients enrolled in a web-based multimedia education and information program had significantly lower anxiety scores compared to controls. Also, our patient population was informed by a well-experienced nurse who was trained in colonoscopy. This might have decreased the pre-procedural anxiety levels in our patients, which might in turn have influenced the correlation between anxiety and sedation.

This study had some limitations. First, we evaluated a cohort with relatively low levels of anxiety, possibly due to good pre-procedural information. This anxiety level might have decreased the need for sedation, which might have influenced the correlations between anxiety and sedation-related parameters. The results may change in a larger cohort with varied anxiety levels. Second, deep sedation was used. Although this level of sedation might have eliminated the effect of anxiety on drug dosage, it might also have increased the recovery time and postoperative complication rate, which was the case in our study as evidenced by a complication rate of 10.6%. The use of opioids might have decreased the propofol doses and should be considered for longer and more painful procedures with interventions. 

## Conclusion

The pre-procedural anxiety level was not related to propofol requirement, anaesthesia-related parameters, post-procedural recovery, or surgeon and patient satisfaction when using deep sedation. Further studies should evaluate the effects of pre-procedural anxiety levels on the required levels of sedation.

## Figures and Tables

**Table 1.** Patients’ Characteristics and Procedure-Related Parameters

Age (years)	49.1 ± 10.4
Sex (female/male)	34/32
BMI (kg m^−2^)	27.1 ± 4.6
ASA I/II	52/14
Caecal intubation time (minutes)	10.2 ± 5.2
Colonoscopy time (minutes)	16.5 ± 6.7
Diagnosis (n)	
Normal	30
Malignancy	3
Bleeding	11
Diverticulitis	1
Haemorrhoid	12
Other	9

ASA, American Society of Anesthesiologists Classification of Physical Health; BMI, body mass index.

Data are median ± SD and number.

**Table 2.** Anaesthesia-Related Parameters

Anaesthesia time (minutes)	21.8 ± 11
Induction time to reach a BIS value of 60 (seconds)	120 [24-168]
Total propofol dose, mg kg^-1^	3.77 ± 1.2
Pre-procedural anxiety	
STAI-S	33.8 ± 4.75
STAI-T	47.83 ± 5.5
Time needed to regain consciousness (Modified Aldrete Score ≥9) (seconds)	213.7 ± 81.7
Anaesthesia-related complications	
None	59
Hypotension	2
Desaturation SpO_2_ <90	5

BIS, bispectral index; STAI-S, Spielberger State Anxiety Inventory (Data are mean ± SD and median [min–max]); STAI-T, Spielberger Trait Anxiety Inventory (Data are mean ± SD and median [min–max]).

**Table 3.** Total Propofol Dosage Adjusted for Time and Weight and Total TCI Intervention Numbers in Different Levels of STAI-S Scores

	STAI-S <39	STAI-S ≥40	*P*
Propofol doses	4 ± 1.3	3.5 ± 1	.06
TCI intervention numbers	8.2 ± 3.8	7.1 ± 2.5	.75

STAI-S, Spielberger State Anxiety Inventory.
